# Survival benefits of simple versus extended cholecystectomy and lymphadenectomy for patients with T1b gallbladder cancer: An analysis of the surveillance, epidemiology, and end results database (2004 to 2013)

**DOI:** 10.1002/cam4.2989

**Published:** 2020-03-31

**Authors:** Li Xu, Haidong Tan, Xiaolei Liu, Jia Huang, Liguo Liu, Shuang Si, Yongliang Sun, Wenying Zhou, Zhiying Yang

**Affiliations:** ^1^ Department of Hepatobiliary and Pancreatic Surgery China‐Japan Friendship Hospital Beijing China

**Keywords:** cholecystectomy, epidemiology, gallbladder cancer, surgical resection, surveillance

## Abstract

Although guidelines recommend extended surgical resection, radical resection and lymphadenectomy for patients with tumor stage (T)1b gallbladder cancer, these procedures are substantially underutilized. This population‐based, retrospective cohort study aimed to evaluate treatment patterns and outcomes of 401 patients using the US Surveillance, Epidemiology, and End Results (SEER) database from 2004 to 2013. Results showed that median overall survival (OS) was 69 months for lymphadenectomy patients and 37 months for those without lymphadenectomy. Lymphadenectomy also tended to prolong cancer‐specific survival (CSS), although the differences were not statistically significant. OS and CSS were similar for patients who received simple cholecystectomy and extended surgical resection. Cox proportional hazards regression models revealed survival advantages in patients with stage T1bN0 gallbladder cancer compared to those with stage T1bN1, and patients who received simple cholecystectomy plus lymphadenectomy compared to those who did not receive lymph node dissection. In further analyses, patients undergoing simple cholecystectomy who had five or more lymph nodes excised had better OS and CSS than those without lymph node dissection. In conclusion, survival advantages are shown for patients with T1b gallbladder cancer undergoing surgeries with lymphadenectomy. Future studies with longer follow‐up and control of potential confounders are highly warranted.

## Graphical abstract

Our study demonstrated that using extensive lymphadenectomy (≥ 5 lymph nodes) would provide a significant survival benefit. The substantial effort is recommended to made to increase the use of lymphadenectomy, radical resection/radical cholecystectomy, and extended surgical resection in the treatment of gall bladder cancer. However, the survival benefit of extended surgical resection was not significantly better than that of simple cholecystectomy, and could have been affected by few patients who underwent resection and selection bias.

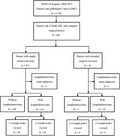

## 1 INTRODUCTION

Prevalence of gallbladder cancer is highest among extrahepatic biliary cancers and is associated with distinct epidemiological features,^1^ including high prevalence among women, particularly in countries with a low sociodemographic index^2^, ^3^, ^4^; and high prevalence rates among American Indians and populations in South America, Asia, and the Middle East.^2^, ^3^, ^5^, ^6^, ^7^ Genetic factors are thought to contribute to increased risk in some of these populations.^8^, ^9^, ^10^

Relying on the presentation and stage of disease, the prognosis of patients with gallbladder cancer varies greatly. Gallbladder cancer is staged using the Tumor, Node, and Metastasis (TNM) system. The T stage of the TNM system (8th edition) is based on tumor invasion into the lamina propria (T1a) or muscular layer (T1b). Nodal stages are classified as N1 or N2 if up to three or four or more nodes are positive, respectively, and the metastasis classification has 1 stage for any metastasis to tissues beyond the lymph nodes. Studies in both high‐ and low‐incidence countries have demonstrated decreases in 5‐year survival rates from 90% in early‐stage disease to 1% in advanced disease.^11^, ^12^, ^13^, ^14^, ^15^

Optimal management of all stages of gallbladder cancer relies heavily on surgical resection. For patients with advanced‐stage disease (II, III, or IV), evidence accumulated over the past two decades indicates that the gallbladder and surrounding structures should be removed.^16^, ^17^, ^18^ An extended surgical resection (ESR) removes the gallbladder and liver, whereas a radical resection (RR) or radical cholecystectomy (RC) removes the gallbladder, liver, regional lymph nodes, and, optionally, the bile duct. For treatment of early‐stage disease, the development of recommendations has been hampered by the inclusion of a heterogeneous group of tumors as stage I and the lack of consistency in the definitions of ESR and RR/RC. This problem was addressed in studies conducted in the United States of America (USA) and Asia that examined patient outcomes associated with stages T0, T1a, and T1b gallbladder cancer. Results of those studies suggested that simple cholecystectomy that removed only the gallbladder was the optimal treatment for patients with T0 or T1a gallbladder cancer.^19^, ^20^, ^21^, ^22^, ^23^ However, simple cholecystectomy has not always provided as much benefit as ESR or RR/RC for patients with T1b gallbladder cancer.^18^, ^19^, ^20^, ^21^, ^22^ Guidelines of the National Comprehensive Cancer Network (NCCN) suggest that controversy exists surrounding benefits of radical resection vs simple cholecystectomy in treatment of T1b tumors.^24^, ^25^

Despite the fact that NCCN guidelines have recommend the use of ESR or RC for T1b‐T4 gallbladder cancer, studies that have used population‐based data from the Surveillance, Epidemiology, and End Results (SEER) database, published as recently as 2013, demonstrate that extended resection and lymphadenectomy continue to be underutilized in treating early‐stage gallbladder cancer.^14^, ^26^, ^27^The failure to follow guidelines for early‐stage gallbladder cancer may have resulted from past controversies regarding the benefits of ESR or RC versus simple cholecystectomy. Thus, it is crucial to continue to gather data on the treatment for early‐stage gallbladder cancer. However, recent studies of gallbladder cancer data in the SEER database are lacking.

To fill this knowledge gap, this study analyzed population‐based SEER data from 2004 to 2013 to investigate optimal surgical selection, clinical benefits of lymphadenectomy, and decisions regarding which lymph nodes should be excised in patients with T1b gallbladder cancer.

## 2 PATIENTS AND METHODS

### 2.1 Data source

The SEER database was established in 1973 by the National Cancer Institute of the National Institutes of Health Surveillance Research Program with the goal of collecting cancer statistics in the US. The continuous database currently includes patient data from 18 regional registries that track almost one‐third of the US population. Data submitted to SEER include patient demographics and morphological and histological codes, TNM stage, tumor histology, tumor size, and patient survival.^28^

### 2.2 Study population

In this population‐based, retrospective cohort study, we assessed outcomes of patients diagnosed with primary T1b gallbladder cancer and whose records were submitted to SEER between 2004 and 2013. T1b gallbladder cancer was defined using the criteria of the International Classification of Diseases for Oncology version 3 (ICD‐O‐3) code C23.9. The following histological types were included: adenocarcinoma (code 8140‐8147), papillary carcinoma or papillary adenocarcinoma (codes 8050‐8052 and 8260‐8263), signet ring cell carcinoma (code 8490), small cell carcinoma (codes 8040‐8046), carcinoma not otherwise specified (codes 8010‐8015), and undifferentiated carcinoma (codes 8020‐8022). Patients with any other histological type of gallbladder cancer, patients with a prior or secondary cancer, and patients whose records lacked information on the type of surgery were excluded.

### 2.3 Outcomes and variables

Major outcomes of this study were overall survival (OS) and cancer‐specific survival (CSS). Demographic covariates included age, sex, marital status, and race/ethnicity. Marital status was categorized as single, married, and separated/divorced/widowed, while race was categorized as white, black, and other. Clinical covariates are node (N) stage, categorized as N0 and N1 as classified by the American Joint Committee on Cancer (AJCC) (6th edition); tumor histology, which is categorized as adenocarcinoma, papillary or papillary adenocarcinoma, and others; and tumor grade, which is categorized as well differentiated, moderately differentiated, and poorly differentiated or undifferentiated. Independent variables consisted of surgical approach (simple vs extended cholecystectomy) and number of lymph nodes examined. Extended surgical resection/extended cholecystectomy (ESR) was defined in the present study as cholecystectomy accompanied by any type of liver resection, including lobectomy. This definition has been used in previous analyses of the SEER database.^18^

### 2.4 Statistical analysis

Continuous variables are indicated as means and standard deviations (SD); categorical variables are presented as numbers (n) and percentages (%). The comparison of OS and CSS between groups was conducted using Kaplan‐Meier method with log‐rank test. The treatment effect of the type of surgery on OS and CSS, after adjusting for age, gender, marital status, race, clinical N stage, histological type of tumors, and differentiation were analyzed using multivariate Cox proportional hazards regression analysis. Results are represented as adjusted hazard ratios (aHR) with corresponding 95% confidence intervals (CI) and P values. All P values were two‐sided, and *P* < .05 was considered statistically significant. All statistical analyses were performed using the statistical software package Statistical Product and Service Solutions (SPSS) IBM version 22 (IBM, Armonk, NY, USA).

## 3 RESULTS

The SEER‐18 registry contains data from 8720 patients with gallbladder cancer whose records were submitted by US participating hospitals from 2004 to 2013. We limited our analyses to the data of 401 patients with T1bM0 GBC whose records had complete information on patients’ surgical history. Clinical and demographic characteristics for these patients are shown in Table 1. The mean age of all eligible patients was 70.1 years and about three‐quarters were female (74.3%). Whites constituted 75.1% of the patients; 12.1% were black, and 12.8% were of other ethnicities. Almost all the patients had clinical N0 stage (92.2%). The most prevalent subtype of gallbladder cancer was adenocarcinoma (74.3%). The most common differentiation of the tumors was moderately differentiated (53.0%), followed by well differentiated (27.6%), and poorly differentiated (17.8%; Table 1).

**Table 1:** Demographics and clinicopathological features of patients included in this study

Variable	N = 401, n (%)
Age (y)	70.12 ± 13.03^a^, ^a^
Gender	
Male	103 (25.7)
Female	298 (74.3)
Marital status^b^, ^b^	
Single	51 (13.4)
Married	197 (51.8)
Separated/Divorced/Widowed	132(34.8)
Race^c^, ^c^	
White	299 (75.1)
Black	48 (12.1)
Other	51 (12.8)
Clinical N stage^d^, ^d^	
N0	355 (92.2)
N1	30 (7.8)
Histological type	
Adenocarcinoma	298 (74.3)
Papillary or papillary adenocarcinoma	82 (20.4)
Other	21 (5.3)
Differentiation^e^, ^e^	
Well differentiated	102 (27.6)
Moderately differentiated	196 (53.0)
Poorly differentiated	66 (17.8)
Undifferentiated	6 (1.6)
Lymph nodes excision^f^, ^f^	
No	247 (63.2)
Yes	144 (36.8)
Surgery	
Simple cholecystectomy	375 (93.5)
Extended surgical resection	26 (6.5)

^a^ Values are mean ± standard deviation.

^b^ Information on marital status was not available for 21 patients.

^c^ Information on race was not available for 3 patients.

^d^ Information on clinical N stage was not available for 16 patients.

^e^ Information on differentiation was not available for 31 patients.

^f^ Information on lymph nodes excision was not available for 10 patients.

**Figure 1:**
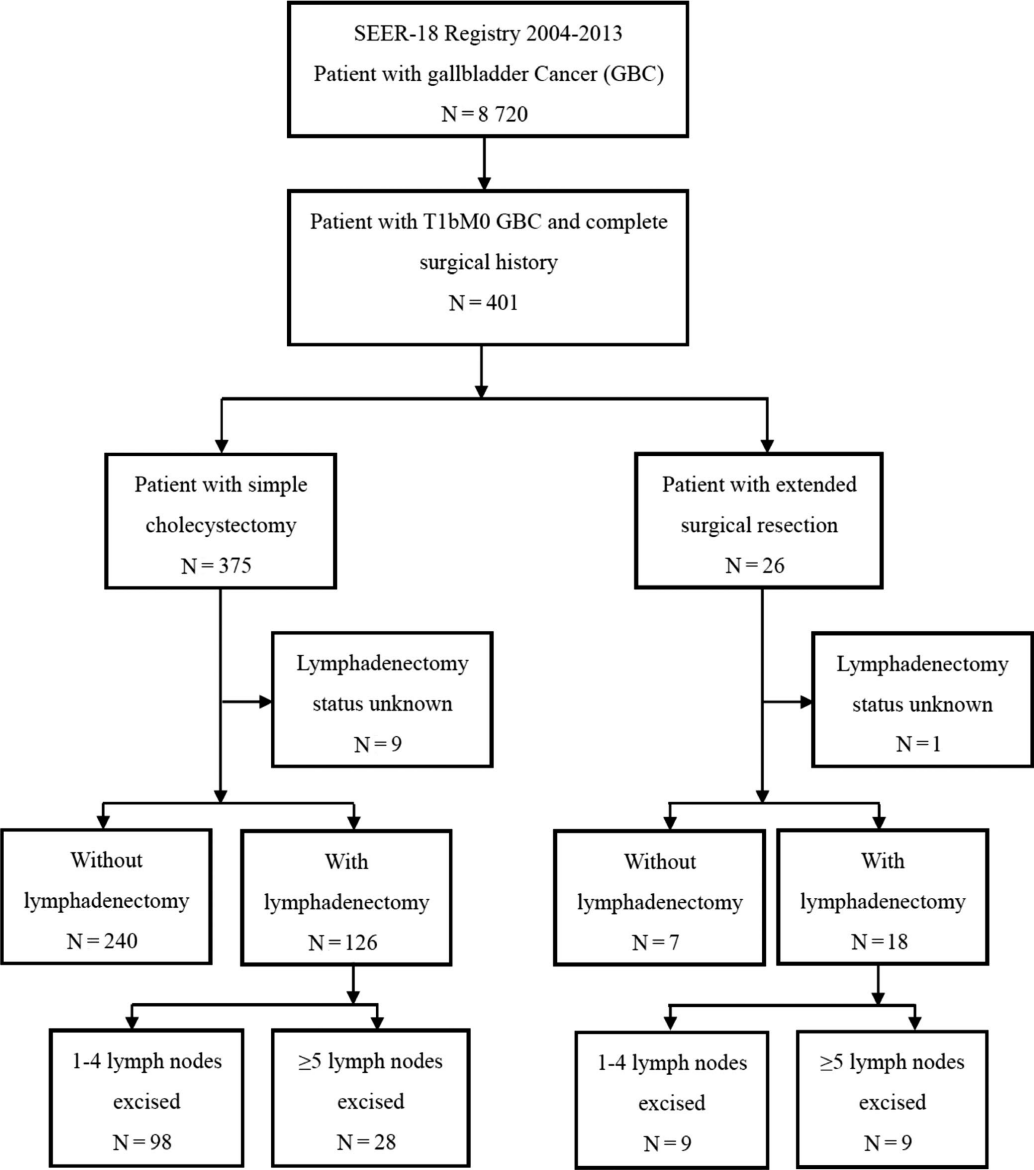
The flow chart of study population. A total of 401 patients with T1bM0 gall bladder cancer constitute the population of this study. Abbreviation: Surveillance, Epidemiology, and End Results (SEER)

Figure 1 illustrates the study flow and process. Among 401 patients, 375 (93.5%) received simple cholecystectomy, and 26 (6.5%) received ESR. Data of 10 patients of unknown lymphadenectomy status were excluded from further analyses. Among patients undergoing simple cholecystectomy, 126 received lymphadenectomy (98 had 1‐4 lymph nodes excised; 28 had 5 or more lymph nodes excised). Among patients undergoing ESR, 18 had lymphadenectomy. A total of 144 patients had lymph node excisions.

A total of 193 deaths occurred during the study period: 64.8% from gallbladder cancer, 13.0% from heart or vascular disease, 5.2% from other types of cancer, 3.1% from chronic obstructive pulmonary disease, 2.1% from an accident, 1.6% from nephrotic disease, 7.7% from other causes, and 2.6% were unknown. The median follow‐up time was 25 months (inter‐quartile range: 10 to 55 months). The 1‐, 3‐, and 5‐year OS rates were 79.7%, 54.6%, and 46.4%, respectively, and the 1‐, 3‐, and 5‐year CSS rates were 83.1%, 64.1%, and 57.1%, respectively.

**Figure 2:**
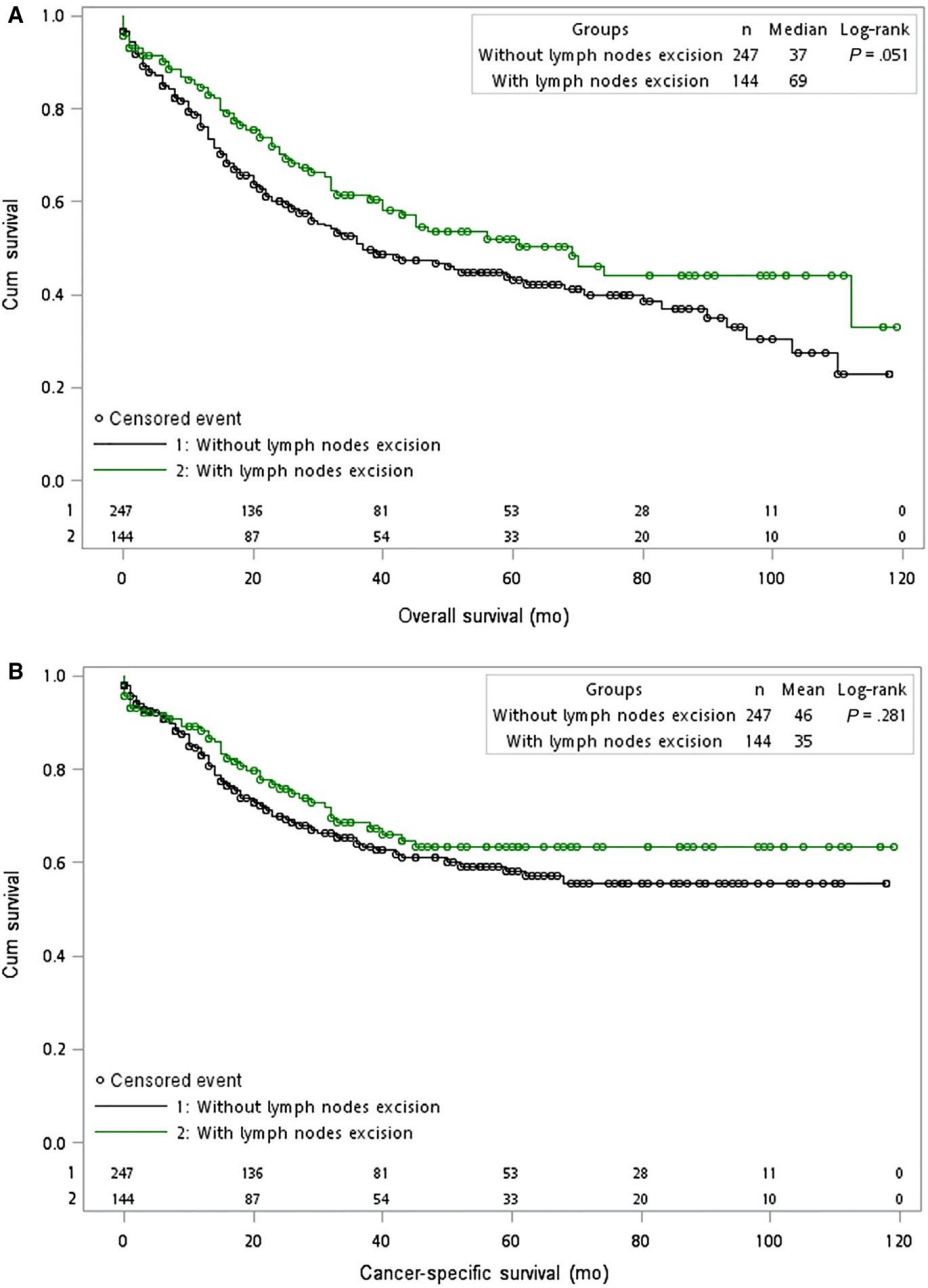
Kaplan–Meier curves for (A) overall survival [OS] and (B) cancer‐specific survival [CSS] in patients with T1b gallbladder cancer between 2004 and 2013, stratified by use of lymphadenectomy (use = green; no use = black). Green or black circles represent censored events. The *x*‐axes show overall survival in months; the *y*‐axes show cumulative survival. The *P*‐values describe the comparison of OS or CSS for patients who did or did not undergo lymphadenectomy. Abbreviations: Cum, cumulative

The median OS time was modestly longer in patients who received lymphadenectomy (69 months) than in those who did not (37 months),but without statistical significance (*P* = .051; Figure 2A). The 1‐, 3‐, and 5‐year OS rates for patients who had 1 or more lymph nodes excised versus no lymph node excised were 84.6%, 61.6% and 52.0% vs 76.2%, 50.9%, and 43.1%, respectively. The median CSS time between patients who did and did not received lymphadenectomy was 46 vs 35 months, *P* = .281 (Figure 2B). The 1‐, 3‐, and 5‐year CSS rates in patients with lymph nodes excision vs those without were 89.1%, 67.9%, 63.1% vs 83.1%, 64.1%, 58.2%, respectively.

**Figure 3:**
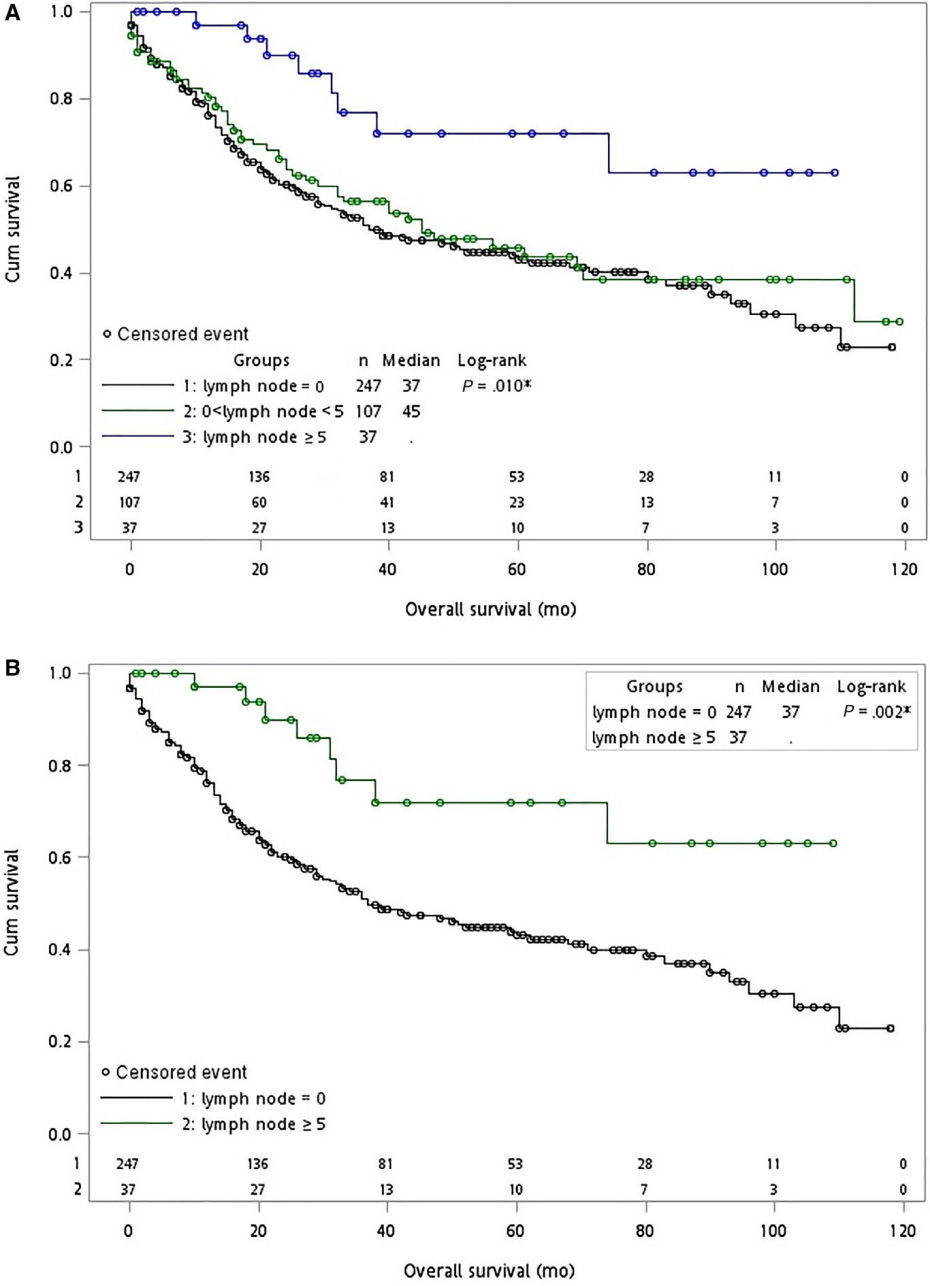
Kaplan–Meier curves for overall survival (OS) in patients with T1b gallbladder cancer between 2004 and 2013. Blue, green or black circles represent censored events. The *x*‐axes show OS in months; the *y*‐axes show cumulative survival. The *P*‐values shown describe the comparison of OS for patients whose lymph nodes = 0, 0 < lymph node < 5, and lymph node ≥5 in (A) three groups and (B) two groups, respectively. Abbreviations: Cum, cumulative

We further compared patients between subgroups according to the number of lymph nodes excised. Among all patients who underwent surgery, the OS was significantly better in those who had more than 5 lymph nodes dissected (*P* = .01, Figure 3A; *P* = .002, Figure 3B).

**Figure 4:**
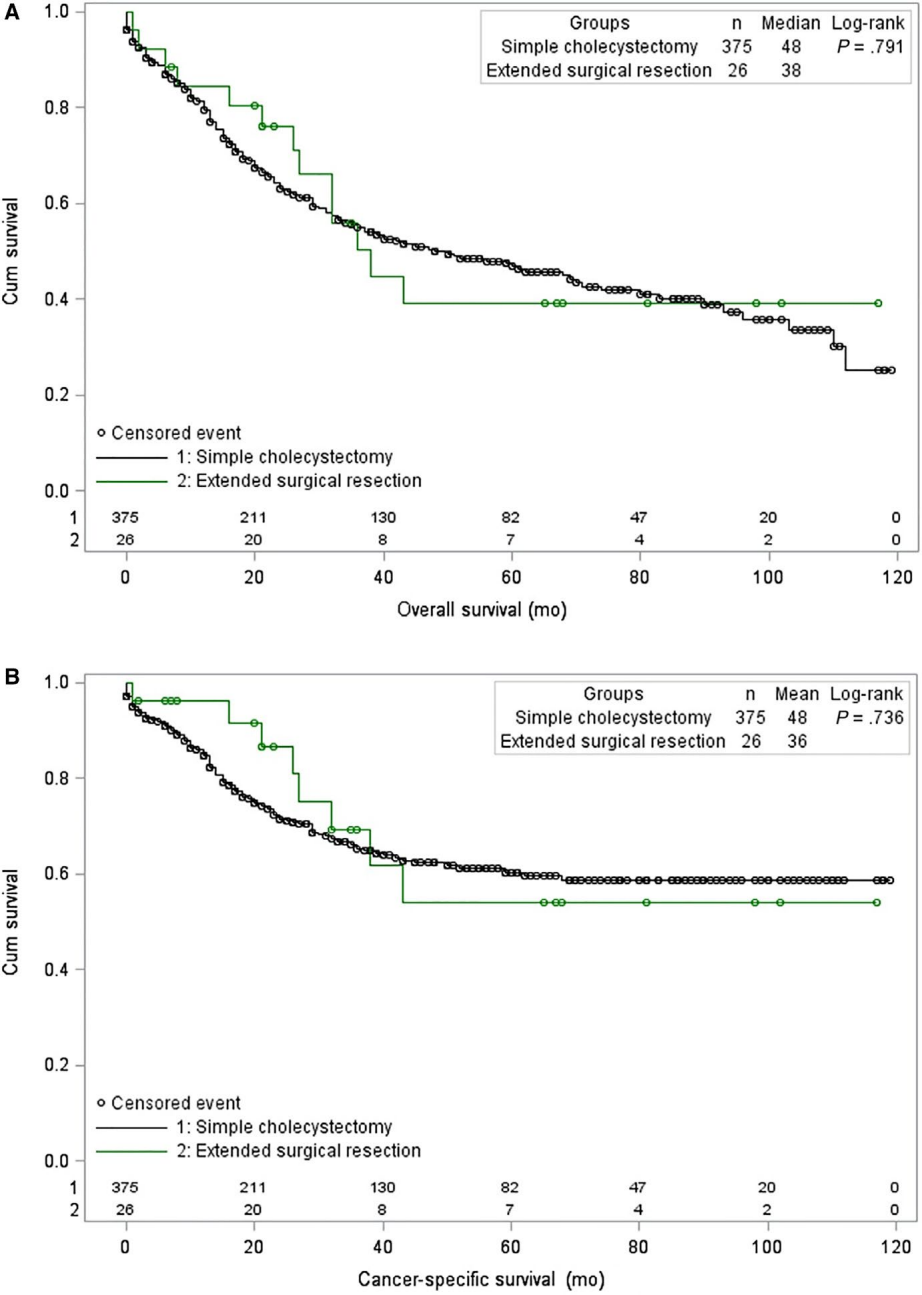
Kaplan–Meier curves for (A) overall survival [OS] and (B) cancer‐specific survival [CSS] in patients with T1b gallbladder cancer between 2004 and 2013, stratified by type of surgery (extended surgical resection = green; simple cholecystectomy = black). Green or black circles represent censored events. The *x*‐axes show overall survival in months; the *y*‐axes show cumulative survival. The *P*‐values shown describe the comparison of OS or CSS for patients who underwent extended surgical resection or simple cholecystectomy. Abbreviations: Cum, cumulative

Figure 4A illustrates that the OS rates were similar between patients who received simple cholecystectomy and those who received ESR (median OS: 48 vs 38 months, *P* = .791). Figure 4B shows that median CSS rates also were similar between patients who received simple cholecystectomy and those who received ESR (48 vs 36 months, *P* = .736). For simple cholecystectomy, the 1‐, 3‐, and 5‐year OS rates were 79.4%, 54.9%, and 46.8%, respectively, and the 1‐, 3‐, and 5‐year CSS rates were 84.7%, 65.3%, and 60.4%, respectively. For ESR, the 1‐, 3‐, and 5‐year OS rates were 84.4%, 50.3%, and 39.1%, respectively, and the CSS rates were 96.2%, 69.4%, and 54.0%, respectively.

The results of Cox proportional hazards regression for OS and CSS among all patients are summarized in Table 2. Significant age differences between OS and CSS suggest that as the age increases, risk will also increase (*P* = .020). Also, women had significantly better OS and CSS than did men (*P* = .012). Differences in OS and CSS between different ethnic groups were not significant. Patients with clinical AJCC N1 stage had significantly higher risk of death (OS and CSS) than those with AJCC N0 stage (both *P* < .001). Furthermore, patients with poorly differentiated or undifferentiated cancers had significantly poorer CSS, but not OS, than did those with well‐differentiated cancers (*P* = .021).

**Table 2:** Multivariate cox proportional hazards regression models of overall survival and cancer‐specific survival of patients with T1b gallbladder cancer (N = 401)

Variable	N	OS		CSS	
		aHR (95%CI)	*P*‐value	aHR (95%CI)	*P*‐value
Age (y)		1.043 (1.027, 1.060)	<.001^a^, ^a^	1.022 (1.003, 1.040)	.020^a^, ^a^
Gender					
Male	103	Reference		Reference	
Female	298	0.612 (0.417, 0.896)	.012^a^, ^a^	0.543 (0.350, 0.844)	.007^a^, ^a^
Marital status^b^, ^b^					
Single	51	Reference		Reference	
Married	197	0.78 (0.464, 1.309)	.346	0.842 (0.464, 1.526)	.57
Separated/Divorced/Widowed	132	0.867 (0.506, 1.486)	.605	0.590 (0.303, 1.150)	.121
Race^c^, ^c^					
White	299	Reference		Reference	
Black	48	1.334 (0.787, 2.261)	.284	1.157 (0.606, 2.211)	.658
Other	51	1.433 (0.876, 2.345)	.152	1.393 (0.754, 2.572)	.29
Clinical N stage^d^, ^d^					
N0	355	Reference		Reference	
N1	30	4.055 (2.186, 7.520)	<.001^a^, ^a^	4.631 (2.284, 9.393)	<.001^a^, ^a^
Histological type					
Adenocarcinoma	298	Reference		Reference	
Papillary or papillary adenocarcinoma	82	0.772 (0.498, 1.196)	.247	0.713 (0.409, 1.243)	.233
Other	21	1.149 (0.567, 2.329)	.701	1.214 (0.568, 2.596)	.617
Differentiation^e^, ^e^					
Well differentiated	102	Reference		Reference	
Moderately differentiated	196	0.982 (0.660, 1.463)	.931	0.997 (0.596, 1.670)	.992
Poorly differentiated/Undifferentiated	72	1.511 (0.934, 2.445)	.093	1.981 (1.109, 3.540)	.021*
Group^f^, ^f^					
Simple cholecystectomy without lymph node excision	240	Reference		Reference	
Simple cholecystectomy with lymph node excision	126	0.630 (0.422, 0.940)	.024^a^, ^a^	0.545 (0.327, 0.909)	.020^a^, ^a^
Extended surgical resection without lymph node excision	7	2.512 (0.899, 7.021)	.079	0.730 (0.100, 5.352)	.757
Extended surgical resection with lymph node excision	18	0.772 (0.332, 1.797)	.548	0.740 (0.289, 1.893)	.529

Abbreviations: aHR, adjusted hazard ratio; CI, confidence interval; CSS, cancer‐specific survival; OS, overall survival.

^a^ Indicates a significant factor, *P* < .05

^b^ Information on marital status was not available for 21 patients.

^c^ Information on race was not available for 3 patients.

^d^ Information on clinical N stage was not available for 16 patients.

^e^ Information on differentiation was not available for 31 patients.

^f^ Information on lymph nodes excision was not available for 10 patients.

When comparing survival for patients who were stratified by type of surgery and use of lymphadenectomy, we used patients who underwent simple cholecystectomy without lymphadenectomy as the reference group. After controlling for potential confounders, we found that patients who underwent simple cholecystectomy with lymph nodes excision had a significant benefit for both OS and CSS (OS: aHR = 0.630, 95%CI: 0.422 to 0.940, *P* = .024; CSS: aHR = 0.545, 95%CI:0.327 to 0.909, *P* = .020). In contrast, neither ESR with or without lymphadenectomy had significantly better OS or CSS than the reference group (Table 2).

**Figure 5:**
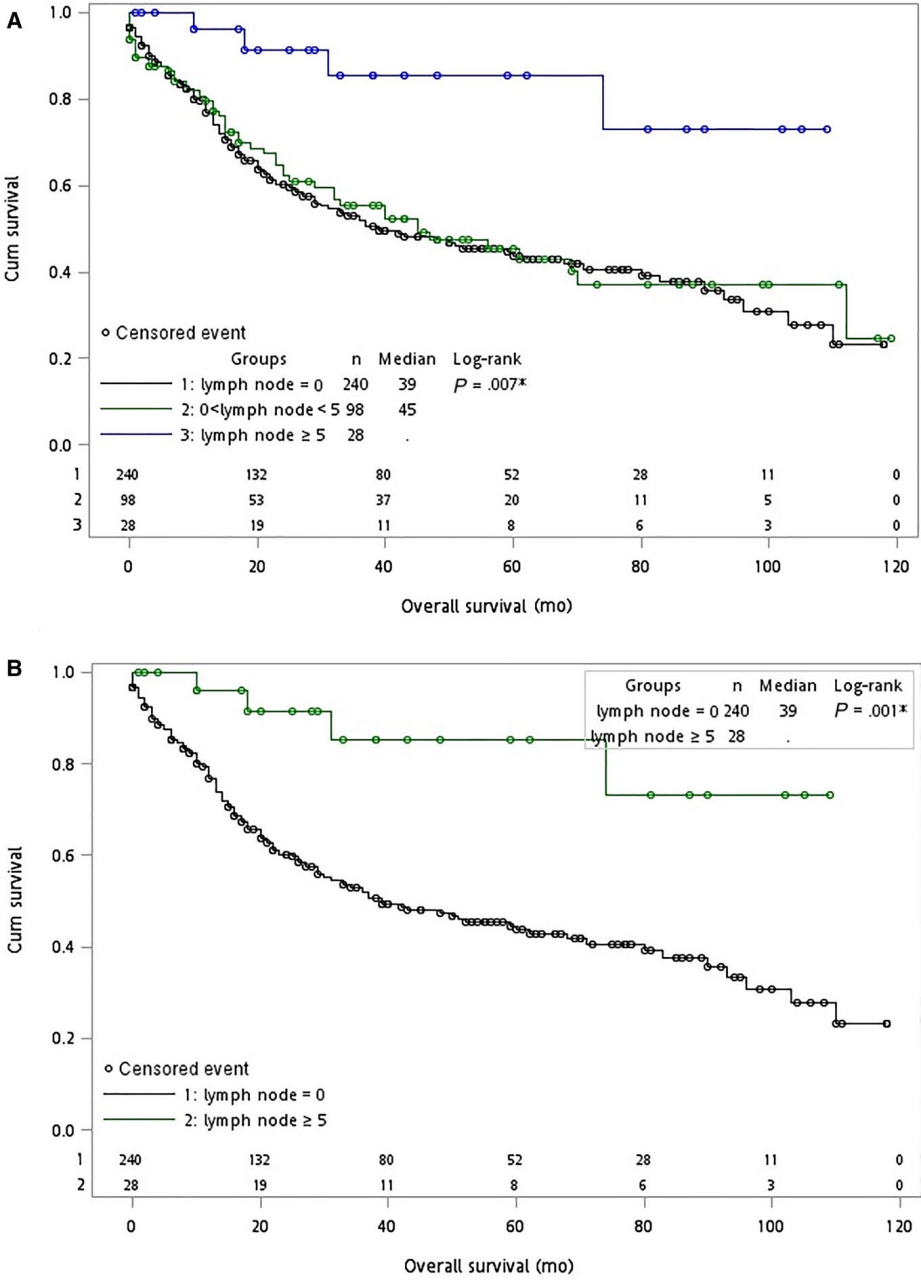
Kaplan–Meier curves for overall survival (OS) in patients with T1b gallbladder cancer between 2004 and 2013. Blue, green, or black circles represent censored events. The *x*‐axes show OS in months; the *y*‐axes show cumulative survival. The *P*‐values shown describe the comparison of OS for patients whose lymph nodes = 0, 0 < lymph node < 5, and lymph node ≥5 in (A) Three group and (B) Two group, respectively. Abbreviations: Cum, cumulative

Table 3 shows the effects on survival of patients undergoing simple cholecystectomy by the number of lymph nodes excised patients were stratified into three categories: without lymph node excision, 1‐4 lymph nodes excised, and ≥ 5 lymph nodes excised as described previously.^29^ Patients who had 5 or more lymph nodes excised showed a significant advantage in survival as compared to no lymph node dissection. For OS, aHR = 0.231, 95%CI: 0.085‐0.627; *P* = .004; for CSS, aHR = 0.183, 95%CI: 0.045‐0.744; *P* = .018. However, the survival advantages for 1‐4 lymph nodes excised were not significant. The survival curves are shown in Figure 5. The groups with ≥ 5 lymph nodes dissected were associated with significant OS advantages (*P* = .007,Figure 5A; *P* = .001, Figure 5B).

**Table 3:** Cox proportional hazards regression model of overall survival and cancer‐specific survival after simple cholecystectomy for patients with T1b gallbladder cancer (N = 366^a^)

Variable	N (%)	OS		CSS	
		aHR (95%CI)^c^	*P*‐value	aHR (95%CI)^c^	*P*‐value
Group (Cut off ≥ 5)					
Simple cholecystectomy without lymph node excision	240	Reference		Reference	
Simple cholecystectomy with 1‐4 lymph nodes excised	98	0.943 (0.676, 1.317)	0.731	0.981 (0.651, 1.478)	0.925
Simple cholecystectomy with ≥ 5 lymph nodes excised	28	0.231 (0.085, 0.627)	0.004^b^	0.183 (0.045, 0.744)	0.018^b^
Group(Cut off ≥ 4)					
Simple cholecystectomy without lymph node excision	240	Reference		Reference	
Simple cholecystectomy with 1‐3 lymph nodes excised	93	0.955 (0.681, 1.340)	0.792	0.963 (0.633, 1.466)	0.861
Simple cholecystectomy with ≥ 4 lymph nodes excised	33	0.299 (0.132, 0.679)	0.004^b^	0.322 (0.118, 0.879)	0.027^b^
Group(Cut off ≥ 6)					
Simple cholecystectomy without lymph node excision	240	Reference		Reference	
Simple cholecystectomy with 1‐5 lymph nodes excised	103	0.873 (0.625, 1.219)	0.425	0.917 (0.609, 1.382)	0.679
Simple cholecystectomy with ≥ 6 lymph nodes excised	23	0.303 (0.112, 0.821)	0.019^b^	0.231 (0.057, 0.938)	0.040^b^

Abbreviations: aHR, adjusted hazard ratio; CI, confidence interval; CSS, cancer‐specific survival; HR, hazard ratio; OS, overall survival.

^a^ Nine out of 375 patients, who underwent simple cholecystectomy and had unknown number of lymph nodes excised, were excluded. Thus, a total of 366 patients was included in this analysis.

^b^ Indicates a significant factor, *P* < .05

^c^ Model was adjusted by all variables.

## 4 DISCUSSION

The present population‐based study, revealed that performing lymph node dissection in patients with T1b gall bladder cancer, specifically with excision of more than five lymph nodes, provides a significant benefit in OS and CSS, but that extended surgical resection does not appear to increase survival significantly. Currently, surgical treatment is recognized as the only curative choice for gallbladder cancer, which known to be especially aggressive and associated with poor prognosis. Previous studies of the SEER database have revealed a continuing trend of underutilization of ESR and RR/RC for patients with T1b gallbladder cancer,^14^, ^26^, ^27^ a population of patients for whom the optimal type of surgery had been controversial.^24^, ^25^ Despite this troubling finding, the issue has not been revisited for several years. Consequently, our present retrospective cohort study used the SEER database to evaluate the comprehensive records of 401 patients with T1b gallbladder cancer diagnosed from 2004 to 2013. Results of our study revealed that only 38.4% of gallbladder cancer patients have lymph nodes excised, suggesting continued insufficient use of lymphadenectomy. Although the use of lymphadenectomy is still unsettled, our present analysis supports the continued and expanded use of extensive lymphadenectomy for patients with T1b gallbladder cancer.

We believe that the low rate of lymphadenectomy translates to poorer outcomes for many patients, for several reasons. First, we found a significantly longer OS among patients who underwent lymphadenectomy than in those without lymphadenectomy. Second, hazard ratios indicating risk for mortality are higher for OS and CSS in gallbladder cancer patients with clinical stage T1bN1 than in those with clinical stage T1bN0. Third, the CSS was comparable between patients with or without lymphadenectomy. This result may be at least in part because lymphadenectomy was performed only if the lymph node involvement was suggested by preoperative imaging and/or intraoperative exploration of suspected enlarged lymph nodes. On the other hand, the actual pathological stage N of un‐resected lymph nodes could not be obtained due to the limitation of SEER database and retrospective nature of the study. Thus, more N0 cases may have been included among the patients without lymphadenectomy than in those with lymphadenectomy, which may have affected the results of the CSS. However, taken together, this study repeatedly demonstrated the benefit of lymphadenectomy in T1b gallbladder cancer patients.

In 2009, Jensen et al^30^ reported that lymph node evaluation was conducted on only half of patients who underwent RR/RC and less than one‐third of patients who underwent simple cholecystectomy. While it was known in such earlier studies that lymphadenectomy provided benefits for patients with gallbladder cancer, underutilization was acknowledged as a persistent problem. Tran et al^15^ reported that from 1988 to 1992, 72% of patients did not receive lymphadenectomy, and this rate fell to 53% from 2007 to 2009. However, Mayo et al^31^ reported that the proportion of patients receiving lymphadenectomy increased from 28% (1991‐ 1995) to 40% (2003‐2005). Interestingly, Lee et al^32^ evaluated 141 Korean patients diagnosed with T1b gallbladder cancer, and reported that only 13.5% of patients received simple cholecystectomy plus lymphadenectomy. This value is substantially less than the 36% that we documented for patients whose records were deposited in the SEER database. Given that substantial outcome benefits are shown for lymphadenectomy,^30^, ^31^, ^33^, ^34^, ^35^, ^36^ we suspect that the US is not the only country in which outcomes could be substantially improved by performing lymphadenectomy in all patients with T1b gallbladder cancer who were undergoing simple cholecystectomy. Researchers have evaluated the number of lymph nodes excised and its association with survival from gallbladder cancer. Downing et al^29^ reported a survival advantage associated with excision of 1‐4 lymph nodes in patients with incidentally discovered gallbladder cancer, compared to those with no excised lymph nodes, and a survival advantage was also found in patients with 5 or more nodes excised compared to patients with 1‐4 excised nodes. In the present study, we also found that excision of 5 or more lymph nodes offered a significant survival benefit over those who with only 1‐4 lymph nodes excised.

The low rate of ESR in patients included in the present study confirms earlier reports of an underutilization of ESR or RR/RC among patients with T1b in the US.^14^, ^26^, ^27^ Of the 401 patients we evaluated, only 6.5% received an ESR. A similar low rate (4.5%) was found in the report of Jensen and colleagues in the study period 1988‐2004,^21^ and 6% was reported by Hari et al^26^ for the study period 1988‐2008. Mayo et al,^27^ who used data from both SEER and the US Medicare system, determined that 8.9% of patients with T1b gallbladder cancer who were diagnosed between 1991 and 2005 received either RR or hepatectomy. A low rate of using ESR and RR/RC for patients with T1b gallbladder cancer may have contributed to lack of significant change in the risk of cancer‐specific deaths for patients with localized gallbladder cancer during the periods 2001 to 2004, 2005 to 2008, and 2009 to 2012.^37^ The SEER database possibly may not be an adequate representation of the true use of ESR or RR/RC among cancer care facilities in the US. However, this does not appear to be true because a study of the National Cancer Database from 1998 to 2012 reported that just 9% of over 19 000 patients with stage T1 to T4 underwent radical cholecystectomy, while 70% underwent total cholecystectomy.^38^ Further studies of the National Cancer Database and other large registries, such as the California Cancer Registry, should be conducted to confirm results of the present study in patients with T1b gallbladder cancer.

Analysis of the SEER database provides a crucial tool by which to compare trends in gallbladder cancer management in the US with trends in other countries. Lee et al^32^ performed a national study of treatment patterns in Korea and found that 36% of patients with T1b gallbladder cancer received RR/RC. A similar rate of ESR (31%) was found by Goetze et al^39^ among patients in the German Registry. Meta‐analyses of more than 20 studies of populations of several countries have also found that rates of ESR are generally more than 25% for early stage gallbladder cancer.^40^, ^41^ The pronounced differences found in US values for ESR usage suggests that more work should be done to reach international consensus regarding the best surgical approach for T1b gallbladder cancer.

Efforts should also be made to determine if the low rate of ESR and RR/RC in the United States arises from a lack of confirmed benefit to patients. In the present study, results for OS and CSS were similar between patients receiving simple cholecystectomy and those receiving ESR, suggesting perhaps that these results may have been affected by the low number of patients who underwent ESR in this study, and that the survival of patients with T1b who received ESR is lower than it could be due to selection of patients perceived as having a poor prognosis. In a previous SEER study by Downing et al^29^ (2011), a comparison of simple cholecystectomy to ESR for T1b gallbladder cancer also found that ESR had no outcome benefit compared to simple cholecystectomy. A meta‐analysis of the many small studies conducted world‐wide have yielded comparable results, including Lee et al^41^ who found no significant differences in risk ratios between patients undergoing simple cholecystectomy and those undergoing ESR. However, Hari et al^26^ reported that patients who received RR/RC had significantly higher survival rates than those who received a simple cholecystectomy (simple cholecystectomy and ESR were not compared). Until this issue is explored further with randomized controlled trials, we support the increased use of ESR or RR/RC.

The 8th edition of AJCC classifies T2 gallbladder cancer as T2a (stage IIA) and T2b (stage IIB) according to tumor location on either the peritoneal side of the gallbladder or the hepatic side.^42^ Studies show that prognosis is poorer on the hepatic side of the gallbladder than on the peritoneal side.^43^, ^44^ On the other hand, whether the location of T1b gallbladder cancer also influences the outcome of surgical resection remains to be investigated. Since the location of T1b gallbladder is not available in the SEER database, further large‐scaled cohort studies are warranted to explore the effects of T1b gallbladder cancer location on the outcomes of different surgical treatments.

This study has several limitations. First, the SEER database only provides information related to the first course of treatment (treatment within 60 days of diagnosis), which for gallbladder cancer would include surgery and/or radiation. As a result, we were unable to determine the effects of later treatments, including chemotherapy. Secondly, as SEER is a registry of the United States and the majority of patients are Caucasian, our findings may not be applicable to other races or ethnicities, particularly Asians. Thirdly, the SEER database did not record information about patients’ co‐morbidities, life‐style factors or adjuvant chemotherapy, and therefore these factors could not be accounted for in the analytic process, which may have confounded the final results. Other information also may be lacking, such as key operative data and possibly other cancers or patient information that would likely have been useful if known. Finally, the exclusion of cases associated with incomplete records may have introduced selection bias in the included patient cohort. We should also acknowledge that studies examining secondary data retrospectively, such as in this study, are of lower quality than are randomized clinical trials. We urge that clinical trials or large‐scale cohort studies are conducted to confirm our results.

In conclusion, patients treated surgically for T1b gallbladder cancer have survival advantages, and significant benefit is shown for those with extensive lymphadenectomy (>5 lymph nodes excised).While the survival benefit of extended surgical resection is not significantly better than that of simple cholecystectomy, OS is significantly longer in patients receiving simple cholecystectomy with the excision of ≥ 5 lymph nodes than in those with fewer nodes excised. Patients with stage T1bN1 gallbladder cancer have poorer survival and consequently increased risk of death than do those with stage T1bN0 gallbladder cancer. We suggest that substantial effort should be made in the comprehensive treatment of patients with gall bladder cancer to (a) improve adherence to NCCN guidelines and (b) increase the use of lymphadenectomy, radical resection/radical cholecystectomy, and extended surgical resection. Future studies with longer follow‐up and control of potential confounders are highly warranted to confirm results of the present study.

## 5 ETHICS STATEMENT

The SEER registry only provides de‐identified data and, therefore, approval by the Institutional Review Board (IRB) approval was not needed for the present study. Also, because SEER patient data are de‐identified, signed informed consent of patients was waived for this study.

## CONFLICT OF INTERESTS

The authors declared no conflicts of interest.

## AUTHOR CONTRIBUTIONS

Li Xu: Conception and design; Analysis and interpretation of data; literature research; Drafting of the manuscript; Final approval of the manuscript. Haidong Tan, Xiaolei Liu, Jia Huang, Liguo Liu, Shuang Si, Yongliang Sun, Wenying Zhou: Acquisition of data;Final approval of the manuscript; Statistical analysis; Literature research. Zhiying Yang: Conception and design; Critical revision of the manuscript; Final approval of the manuscript.

## Data Availability

The Surveillance, Epidemiology, and End Results (SEER) database (2004‐2013) was utilized in the present study (https://seer.cancer.gov/).
